# CircTRRAP Knockdown Has Cardioprotective Function in Cardiomyocytes via the Signal Regulation of miR-370-3p/PAWR Axis

**DOI:** 10.1155/2022/7125602

**Published:** 2022-02-15

**Authors:** Yuan Zhang, Zhenggong Li, Jiao Wang, Hao Chen, Rui He, Hongkun Wu

**Affiliations:** Department of Cardiology Center, Chongqing General Hospital, Chongqing City 400016, China

## Abstract

**Background:**

Circular RNA Transformation/Transcription Domain Associated Protein (circTRRAP, hsa_circ_0081241) was abnormally upregulated in acute myocardial infarction (AMI) patients. However, its biological role and functional mechanism in AMI remain to be researched.

**Methods:**

Human cardiomyocyte AC16 was exposed to hypoxia to induce cell injury. Cell viability was detected through Cell Counting Kit-8. CircTRRAP, microRNA-370-3p (miR-370-3p), and Pro-Apoptotic WT1 Regulator (PAWR) levels were assayed by reverse transcription-quantitative polymerase chain reaction. Cell proliferation analysis was performed via 5-ethynyl-2′-deoxyuridine (EdU) assay. Cell apoptosis was assessed using flow cytometry and caspase-3 activity assay. The protein levels were measured through western blot. Enzyme-linked immunosorbent assay was used to examine the release of inflammatory cytokines. Oxidative stress was assessed by the commercial kits. Dual-luciferase reporter assay, RNA immunoprecipitation, and RNA pull-down assays were performed for the validation of target interaction.

**Results:**

CircTRRAP was highly expressed following hypoxia treatment in AC16 cells. Downregulation of circTRRAP promoted cell growth but inhibited apoptosis, inflammation, and oxidative stress in hypoxic cells. CircTRRAP could target miR-370-3p, and the regulatory effects of circTRRAP on the hypoxic cells were associated with the sponge function of miR-370-3p. PAWR served as the target for miR-370-3p, and it was regulated by circTRRAP/miR-370-3p axis. The protective role of miR-370-3p was achieved by downregulating the PAWR expression in hypoxia-treated AC16 cells.

**Conclusion:**

These findings demonstrated that silence of circTRRAP exerted the protection against the hypoxia-induced damages in cardiomyocytes through regulating the miR-370-3p and PAWR levels.

## 1. Introduction

Acute myocardial infarction (AMI) is an emergency incident with the sudden and persistent loss of oxygen supply or increase of oxygen demand in the heart [1, 2]. AMI leads to geometric, molecular, structural, and functional changes to evoke ventricular remodeling, and inflammatory response is triggered after myocardial injury [3]. Although the substantial improvement of prognosis has been achieved over the past decades, AMI is still an important cause of morbidity and mortality in human beings worldwide [4]. In addition, AMI has also become a potential threat to young individuals [5]. It remains crucial to explore novel biomarkers in the pathological process of AMI.

Noncoding RNAs with high specificity and sensitivity have been used as biological targets for clinical diagnosis and surveillance of AMI [6, 7]. Circular RNAs (circRNAs) are regulators of genes by depending on the “sponge-like” function of microRNAs (miRNAs), then affecting the disease development in life processes [8]. Knockdown of circROBO2 inhibited apoptosis of cardiomyocytes in AMI through decreasing the level of TRADD by the upregulation of miR-1184 [9]. Overexpression of circCDYL sponged 4793-5p to induce the APP downregulation, thus promoting the proliferation ability of myocardial cells [10]. Zhao et al. reported that circMACF1 suppressed the AMI progression via targeting the miR-500b-5p/EMP1 network [11]. Circular RNA Transformation/Transcription Domain Associated Protein (circTRRAP, hsa_circ_0081241) originates from TRRAP gene, and it was upregulated in AMI patients [12]. The exploration of function and mechanism for circTRRAP in AMI has never been performed.

Zhang et al. found that microRNA-370-3p (miR-370-3p) protected cardiomyocytes from hypoxic injury by targeting TRAF6, and circ_0010729 facilitated the hypoxia-induced dysfunction through regulating the miR-370-3p/TRAF6 axis [13]. Chai et al. stated that Pro-Apoptotic WT1 Regulator (PAWR) was highly expressed in AMI patients, and circ_0068655 contributed to cell damages in hypoxic cardiomyocytes via the mediation of miR-498/PAWR signal axis [14]. The regulatory relation among circTRRAP, miR-370-3p, and PAWR is unclear in the regulation of AMI progression.

Herein, we hypothesized that circTRRAP played a sponge role for miR-370-3p, and PAWR served as a target for miR-370-3p. Furthermore, whether PAWR level could be affected by circTRRAP via targeting miR-370-3p was analyzed. This study performed the research of function and mechanism of circTRRAP in hypoxia-induced cardiomyocyte injury *in vitro*.

## 2. Materials and Methods

### 2.1. Cell Culture and Hypoxic Treatment

Human ventricular cardiomyocyte cell line (AC16; Millipore, Billerica, MA, USA) was cultured in humid condition with 5% CO_2_ at 37°C. Cell medium was produced by adding 10% (*v*/*v*) fetal bovine serum (FBS; Sigma-Aldrich, St. Louis, MO, USA) and 1% (*v*/*v*) penicillin-streptomycin reagent (Sigma-Aldrich) into Dulbecco's modified eagle medium (DMEM; Sigma-Aldrich). The third passage of AC16 cells was used in the study. For hypoxic treatment, cells were incubated in the incubator containing 94% N_2_, 5% CO_2_, and 1% O_2_ for increasing times (0 h, 12 h, 24 h, 48 h).

### 2.2. Cell Transfection

AC16 cells were transiently transfected plasmids or RNAs through Lipofectamine™ 3000 Kit (Invitrogen, Carlsbad, CA, USA). Short interfering RNA (siRNA) or control for circTRRAP (si-circTRRAP, si-NC), mimic or control for miR-370-3p (miR-370-3p, miR-NC), and inhibitor or control for miR-370-3p (in-miR-370-3p, in-miR-NC) were directly obtained from GenePharma (Shanghai, China). In addition, the pcDNA expression plasmid (Invitrogen) was cloned with the PAWR sequence, and the novel pcDNA-PAWR (PAWR) plasmid was applied for overexpression of PAWR. The use concentrations were listed as follows: 40 nM siRNA, 40 nM mimic, 20 nM inhibitor, and 2 *μ*g plasmid.

### 2.3. Cell Counting Kit-8 (CCK-8) Assay

AC16 cells in the 96-well plates following different treatment were added with CCK-8 solution (10 *μ*L/well; KeyGEN, Nanjing, China) for 2 h. Then, the absorbance determination at 450 nm was performed on a microplate reader (Bio-Rad, Hercules, CA, USA).

### 2.4. Reverse Transcription-Quantitative Polymerase Chain Reaction (RT-qPCR) Assay

AC16 cells were washed with phosphate buffer solution (PBS, Sigma-Aldrich), and RNA extraction by Total RNA Extractor (Sangon, Shanghai, China) was performed in accordance with the instruction book. ReverTra Ace® qPCR RT Kit (Toyobo, Kita-Ku, Osaka, Japan) was used for reverse transcription, and SYBR® Green Realtime PCR Master Mix (Toyobo) was applied for quantitative reaction. The miRNA quantification was performed using MystiCq® microRNA cDNA Synthesis Mix and MystiCq® microRNA® SYBR® Green qPCR ReadyMix™ (Sigma-Aldrich). Ct values were collected for relative level analysis via the 2^-∆∆Ct^ method. *β*-Actin has been used as the control gene for expression correction of circRNA and mRNA, while U6 was applied to standardize the miRNA level. The specific primers were synthesized by Sangon, and the sequences are exhibited in Table 1.

### 2.5. CircRNA Stability and Localization Assays

For stability detection, circTRRAP and linear TRRAP levels were assayed through RT-qPCR following treatment of RNase R (GENESEED, Guangzhou, China) for total RNA. For localization analysis, RT-qPCR detection for circTRRAP was conducted after isolation of nuclear and cytoplastic RNAs by PARIS™ Kit (Invitrogen). Glyceraldehyde-phosphate dehydrogenase (GAPDH) for cytoplasm and U6 for nucleus served as the control genes, respectively.

### 2.6. 5-Ethynyl-2′-Deoxyuridine (EdU) Assay

5 × 10^4^/well AC16 cells were plated onto the 96-well plates, followed by hypoxia treatment and cell transfection. The treated cells were performed with EdU staining as per the direction of EdU Cell Proliferation Kit (Sangon). The nuclei were stained with diamidine phenylindole (DAPI; Sangon), and cell analysis was performed through the fluorescence microscope (Olympus, Tokyo, Japan), followed by calculating the ratio of EdU-positive cells (EdU + DAPI) in total cells.

### 2.7. Flow Cytometry

6 × 10^4^ AC16 cells were collected for apoptosis detection using Annexin V-fluorescein isothiocyanate (Annexin V-FITC)/Propidium Iodide (PI) Kit (Sangon). Briefly, cells were resuspended in 1 × Binding Buffer and then incubated with 5 *μ*L Annexin V-FITC and 10 *μ*L PI for 15 min. Instantly, cell determination was implemented on the flow cytometer (BD Biosciences, San Diego, CA, USA). The apoptotic rate was indicated as the percentage of Annexin V+/PI- and Annexin V+/PI+ stained cells in total cells.

### 2.8. Western Blot

Total protein acquisition by radioimmunoprecipitation assay (RIPA) buffer (Sangon) was carried out from AC16 cells in line with the user's manual. The protein concentration was tested through BCA Protein Assay Kit (Sangon), and the blotting detection was performed as previously stated [15]. The rabbit primary antibodies for hypoxia-inducible factor 1*α* (HIF1*α*; ab179483, 1 : 1000), B-cell lymphoma-2 (Bcl-2; ab32124, 1 : 1000), Cleaved caspase 3 (ab2302, 1 : 1000), Bcl-2 associated X (Bax; ab32503, 1 : 1000), PAWR (ab92590, 1 : 1000), and *β*-actin (ab213262, 1 : 3000) were commercially purchased from Abcam (Cambridge, UK). Goat Anti-Rabbit IgG H&L (Abcam, ab205718, 1 : 5000) was used as the secondary antibody. The ImageJ software (NIH, Bethesda, MD, USA) was applied for protein level analysis. *β*-Actin acted as the endogenous control for the objective genes.

### 2.9. Caspase-3 Activity Assay

6 × 10^4^ AC16 cells were planted into the 48-well plates, followed by hypoxic treatment and RNA or plasmid transfection. After 24 h, 4 × 10^6^ cells were washed in PBS, and caspase-3 activity was analyzed by Caspase-3 Cellular Activity Assay Kit (Sigma-Aldrich) following the producer's specification.

### 2.10. Enzyme-Linked Immunosorbent Assay (ELISA)

The supernatants of AC16 cells were harvested for the detection of proinflammatory cytokines. The concentrations of Interleukin-1beta (IL-1*β*), Interleukin-6 (IL-6), and tumor necrosis factor-alpha (TNF-*α*) were measured through Human IL-1*β* ELISA Kit, Human IL-6 ELISA Kit, and Human TNF-*α* ELISA Kit (Sangon), respectively.

### 2.11. Oxidative Assay

Oxidative injury was analyzed by the detection of malondialdehyde (MDA), reactive oxygen species (ROS), and superoxide dismutase (SOD). MDA and ROS levels were examined according to the guidelines of MDA Assay Kit (Sigma-Aldrich) and Total Reactive Oxygen Species (ROS) Assay Kit (Invitrogen). SOD activity was determined through the SOD Assay Kit (Sigma-Aldrich) as per the user's manual.

### 2.12. Dual-Luciferase Reporter Assay

The miRNA target for circTRRAP and target gene for miR-370-3p were predicted by CircInteractome (https://circinteractome.nia.nih.gov/) and microT CDS (https://www.biostars.org/p/143874/), respectively. CircTRRAP sequence containing miR-370-3p binding sites (wild-type, WT) or the mutated sequence (mutant-type, MUT) was cloned into the pmirGLO vector (Promega, Madison, WI, USA). Those acquired plasmids were named as circTRRAP WT and circTRRAP MUT. Meanwhile, the luciferase plasmids for PAWR (PAWR 3′UTR WT, PAWR 3′UTR MUT) were constructed by cloning the PAWR sequence into pmirGLO. The cotransfection of each luciferase construct and miR-NC, miR-370-3p or in-miR-NC, and in-miR-370-3p was performed for 48 h in AC16 cells. The activity of luciferase signal was immediately examined through the Dual-luciferase Reporter Kit (Promega).

### 2.13. RNA Immunoprecipitation (RIP) Assay

The binding between circTRRAP and miR-370-3p was determined through Magna RIP RNA-Binding Protein Immunoprecipitation Kit (Millipore). The magnetic beads were enveloped with antibody against Argonaute-2 (Anti-AGO2) or immunoglobulin G (Anti-IgG), then incubated to cell lysates of AC16 at 4°C overnight. The input group (positive group) was set by lacking antibody incubation in beads. RNA complexes on the beads were isolated; then, RT-qPCR detection was performed for circTRRAP and miR-370-3p.

### 2.14. RNA Pull-Down Assay

Biotin-coupled miRNA mimics were transfected into AC16 cells for 48 h. Bio-miR-370-3p-MUT and Bio-NC were applied as the mutant and negative controls for the Bio-miR-370-3p group. Then, cell incubation with MagnaBind™ streptavidin magnetic beads (Thermo Fisher Scientific, Waltham, MA, USA) was conducted at 4°C overnight. The circTRRAP enrichment was detected using RT-qPCR after RNA isolation and reverse transcription.

### 2.15. Statistical Analysis

Data of each experiment were collected from three independent repetitions with three parallels. The mean ± standard deviation (SD) was used to indicate the final data, and statistical analysis was conducted through SPSS 22.0 (SPSS Inc., Chicago, IL, USA). The significant difference (*P* < 0.05) was determined using Student's *t*-test and analysis of variance (ANOVA) followed by Tukey's test.

## 3. Results

### 3.1. Hypoxia Induced the Significant Upregulation of circTRRAP in AC16 Cells

**Figure 1:**
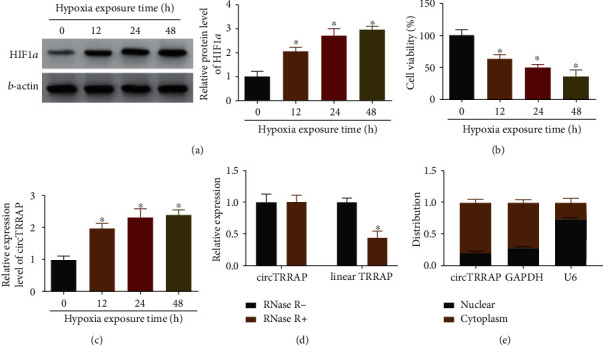
Hypoxia induced the significant upregulation of circTRRAP in AC16 cells. (a) HIF1*α* protein expression was measured by western blot in AC16 cells exposed to hypoxia for 0 h, 12 h, 24 h, or 48 h. (b, c) Cell viability was detected by CCK-8 assay (b), and circTRRAP level was quantified by RT-qPCR (c) in hypoxia-treated AC16 cells. (d, e) The characteristics of circTRRAP were analyzed by stability assay with RNase R treatment (d) and localization assay after cytoplasmic or nuclear RNA isolation (e). The experiments were repeated for three times with three paralleled samples. ^∗^*P* < 0.05.

AC16 cells were exposed to hypoxia condition for 0 h, 12 h, 24 h, and 48 h. HIF1*α* protein expression was much higher in hypoxia treatment groups than that in the control 0 h group, validating the hypoxia induction (Figure 1(a)). CCK-8 results in Figure 1(b) showed that cell viability was reduced after exposure to hypoxia relative to the 0 h group. Interestingly, the relative level of circTRRAP was elevated by hypoxia in a time-dependent way (Figure 1(c)). No conspicuous change of circTRRAP expression was detected in the RNase R- and RNase R+ groups, while linear TRRAP level has been largely downregulated by RNase R treatment (Figure 1(d)). Thus, circTRRAP exhibited higher stability than linear TRRAP in AC16 cells. Additionally, circTRRAP and GAPDH were enriched in the cytoplasm, but U6 was mainly determined in the nucleus of AC cells (Figure 1(e)). The result identified the cytoplasmic localization of circTRRAP in AC16 cells. High expression of circTRRAP was confirmed in hypoxic AC16 cells.

### 3.2. CircTRRAP Knockdown Impeded the Damages Induced by Hypoxia in AC16 Cells

**Figure 2:**
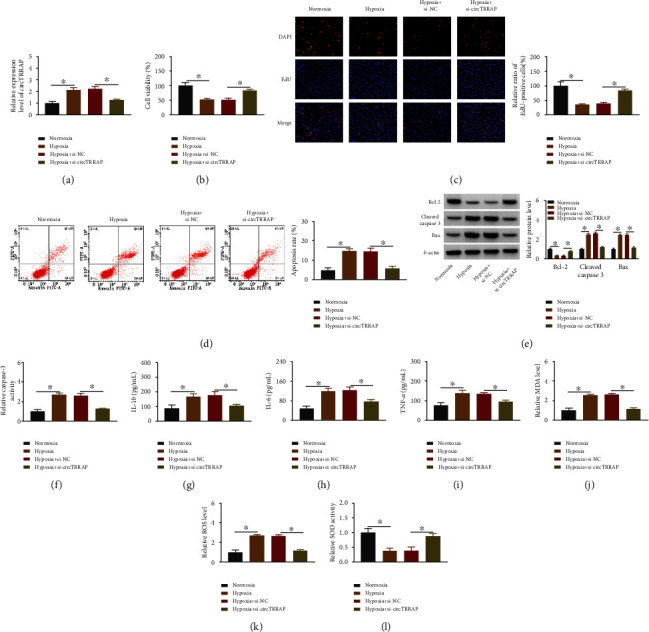
CircTRRAP knockdown impeded the damages induced by hypoxia in AC16 cells. AC16 cells were treated with normoxia, hypoxia (24 h), hypoxia+si-NC, or hypoxia+si-circTRRAP. (a) CircTRRAP expression detection was performed using RT-qPCR assay. (b, c) Cell viability (b) and proliferation (c) analysis was performed using CCK-8 and EdU assays, respectively. (d–f) Cell apoptosis assessment was conducted by flow cytometry for apoptotic rate (d), western blot for apoptotic proteins (e), and caspase-3 assay for caspase-3 activity (f). (g–i) Inflammation evaluation was conducted by ELISA for the concentration detection of IL-1*β* (g), IL-6 (h), and TNF-*α* (i). (j–l) Oxidative injury analysis was performed by MDA (j), ROS (k), and SOD (l) detection. The experiments were repeated for three times with three paralleled samples. ^∗^*P* < 0.05.

Hypoxia-induced upregulation of circTRRAP was attenuated by si-circTRRAP, which demonstrated that the transfection efficiency of si-circTRRAP was obvious (Figure 2(a)). The inhibitory effects of hypoxia on cell viability (Figure 2(b)) and proliferation (Figure 2(c)) were counteracted after cotreatment of hypoxia and si-circTRRAP. The apoptotic rate by flow cytometry manifested that knockdown of circTRRAP partly eliminated the hypoxia-induced apoptotic injury (Figure 2(d)). Furthermore, the apoptosis-related proteins were determined using western blot. The results showed that antiapoptotic Bcl-2 was upregulated while proapoptotic Cleaved caspase 3 and Bax were downregulated in the hypoxia+si-circTRRAP group contrasted to the hypoxia+si-NC group (Figure 2(e)). Downregulation of circTRRAP also abolished the promoting regulation of caspase-3 activity by hypoxia in AC16 cells (Figure 2(f)). In addition, the determination of inflammatory cytokines (IL-1*β*, IL-6, TNF-*α*) suggested that si-circTRRAP inhibited the inflammatory damage in hypoxia-treated AC16 cells (Figures 2(g)–2(i)). Hypoxia induced the oxidative injury to increase the levels of MDA (Figure 2(j)) and ROS (Figure 2(k)) but inhibit the SOD activity (Figure 2(l)); then, si-circTRRAP transfection relieved these influences. Altogether, hypoxia-induced apoptosis, inflammation, and oxidative stress have been partly reversed after inhibition of circTRRAP in AC16 cells.

### 3.3. CircTRRAP Directly Bound to miR-370-3p

**Figure 3:**
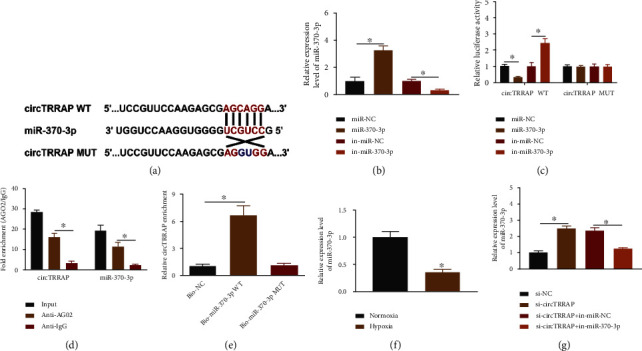
CircTRRAP directly bound to miR-370-3p. (a) Target binding sites between circTRRAP and miR-370-3p were shown by CircInteractome. (b) RT-qPCR was used to analyze the transfection efficiencies of miR-370-3p and in-miR-370-3p. (c–e) Dual-luciferase reporter assay (c), RIP (d), and RNA pull-down assay (e) were applied to confirm the interactive relation between miR-370-3p and circTRRAP. (f) RT-qPCR was used to detect the level of miR-370-3p in normoxia or hypoxia (24 h) treated AC16 cells. (g) The miR-370-3p level was determined through RT-qPCR following transfection with si-NC, si-circTRRAP, si-circTRRAP+in-miR-NC, or si-circTRRAP+in-miR-370-3p. The experiments were repeated for three times with three paralleled samples. ^∗^*P* < 0.05.

The binding sites between circTRRAP and miR-370-3p were displayed by CircInteractome (Figure 3(a)). The results of RT-qPCR assay indicated that miR-370-3p level was evidently increased after transfection of miR-370-3p but repressed by transfection of in-miR-370-3p, compared with the miR-NC or in-miR-NC group (Figure 3(b)). The miR-370-3p overexpression has reduced the luciferase activity, and miR-370-3p downregulation promoted that of the circTRRAP WT group, while no difference of luciferase activity was detected in the circTRRAP MUT group (Figure 3(c)). Meanwhile, the interaction between circTRRAP and miR-370-3p was affirmed through RIP assay (Figure 3(d)) and RNA pull-down assay (Figure 3(e)). The treatment of hypoxia resulted in a suppressive effect on miR-370-3p level in AC16 cells, relative to the normoxia group (Figure 3(f)). Silencing circTRRAP induced the upregulation of miR-370-3p, which was obviously abolished by in-miR-370-3p transfection (Figure 3(g)). Hence, circTRRAP could target miR-370-3p to evoke the negative regulation of miR-370-3p level.

### 3.4. CircTRRAP/miR-370-3p Signal Axis Regulated the Hypoxia-Stimulated Cell Injury

**Figure 4:**
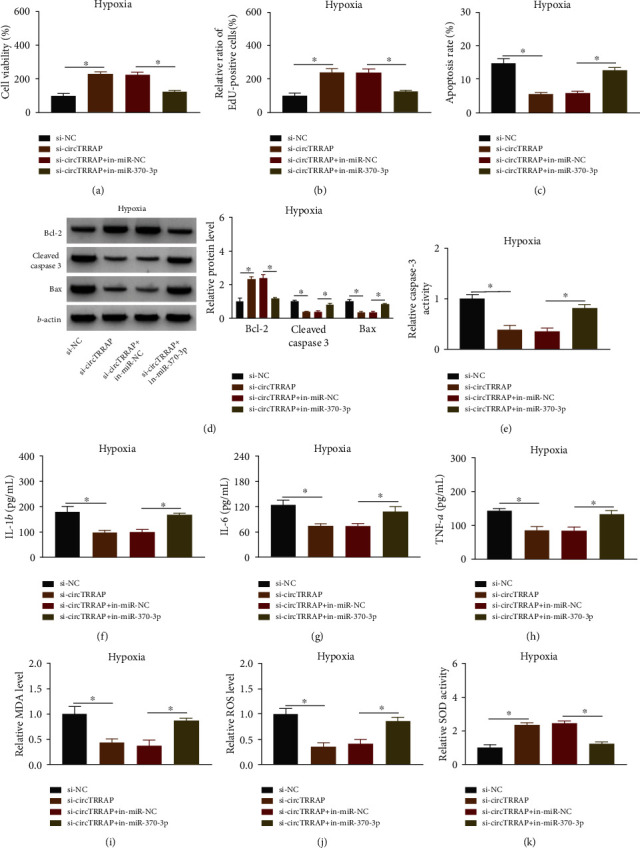
CircTRRAP/miR-370-3p signal axis regulated the hypoxia-stimulated cell injury. AC16 cells with hypoxic treatment (24 h) were transfected with si-NC, si-circTRRAP, si-circTRRAP+in-miR-NC, or si-circTRRAP+in-miR-370-3p. (a, b) Cell viability (a) and proliferation (b) were assessed via CCK-8 assay and EdU assay. (c–e) Cell apoptosis was measured via flow cytometry (c), western blot (d), and caspase-3 activity assay (e). (f–h) Inflammatory response was analyzed via ELISA. (i–k) Oxidative stress was assessed via the commercial kits. The experiments were repeated for three times with three paralleled samples. ^∗^*P* < 0.05.

The regulatory relationship between circTRRAP and miR-370-3p was investigated in hypoxic AC16 cells. The experimental results revealed that miR-370-3p inhibition counterbalanced the stimulative influences of si-circTRRAP on cell viability (Figure 4(a)) and proliferation (Figure 4(b)) but the inhibitory regulation of cell apoptosis (Figures 4(c)–4(e)) in hypoxia-treated AC16 cells. Also, the si-circTRRAP-mediated suppression of inflammatory response (Figures 4(f)–4(h)) and oxidative stress (Figures 4(i)–4(k)) injury was distinctly alleviated following the downregulation of miR-370-3p. These findings elucidated that circTRRAP participated in the regulation of hypoxia-induced cell damages through absorbing miR-370-3p.

### 3.5. PAWR Expression Was Upregulated by circTRRAP via Targeting miR-370-3p

**Figure 5:**
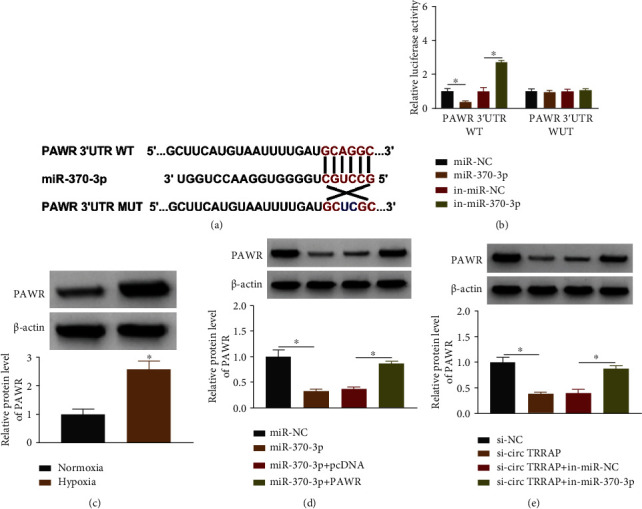
PAWR expression was upregulated by circTRRAP via targeting miR-370-3p. (a) The microT CDS predicted the binding sites between PAWR 3′UTR and miR-370-3p. (b) The combination between miR-370-3p and PAWR 3′UTR was validated through dual-luciferase reporter assay. (c) PAWR protein expression was assayed by western blot in the normoxia or hypoxia group. (d) The protein detection for PAWR was performed using western blot following transfection of miR-NC, miR-370-3p, miR-370-3p + pcDNA, or miR-370-3p + PAWR. (e) Western blot was used for examining the protein level of PAWR in si-circTRRAP, si-circTRRAP+in-miR-370-3p, or the control groups. The experiments were repeated for three times with three paralleled samples. ^∗^*P* < 0.05.

The complementary sites between PAWR 3′UTR and miR-370-3p sequences were exhibited in microT CDS (Figure 5(a)). Then, dual-luciferase reporter assay demonstrated that miR-370-3p could negatively affect the luciferase activity of PAWR 3′UTR WT plasmid but not the PAWR 3′UTR MUT group (Figure 5(b)). The protein level of PAWR was higher in the hypoxia group than that in the normoxia group of AC16 cells (Figure 5(c)). The upregulation of miR-370-3p inhibited the PAWR protein expression, but this inhibition was mitigated through the transfection of PAWR (Figure 5(d)). Moreover, western blot suggested that miR-370-3p inhibitor significantly lightened the si-circTRRAP-induced expression downregulation of PAWR (Figure 5(e)). Collectively, circTRRAP could act as a sponge for miR-370-3p to affect the expression level of PAWR.

### 3.6. Overexpression of miR-370-3p Protected AC16 Cells against the Hypoxia-Induced Injury by Inhibiting PAWR Level

**Figure 6:**
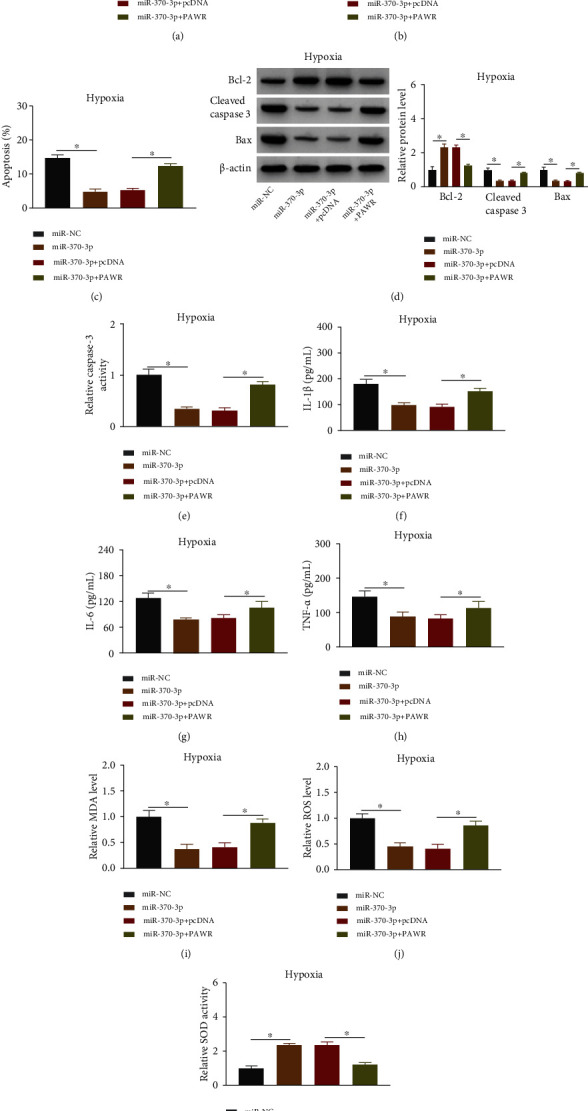
Overexpression of miR-370-3p protected AC16 cells against the hypoxia-induced injury by inhibiting PAWR level. AC16 cells under the hypoxic condition (24 h) were transfected with miR-NC, miR-370-3p, miR-370-3p + pcDNA, or miR-370-3p + PAWR. (a, b) CCK-8 (a) and EdU assay (b) were used to determine cell viability (a) and proliferation (b). (c–e) Flow cytometry (c), western blot (d), and caspase-3 activity assay (e) were applied for apoptosis analysis. (f–h) ELISA was applied for the measurement of inflammatory cytokines. (i–k) The detection kits were used for the evaluation of oxidative stress. The experiments were repeated for three times with three paralleled samples. ^∗^*P* < 0.05.

Furthermore, whether the function of miR-370-3p was related to PAWR was researched in hypoxic AC16 cells. Transfection of miR-370-3p facilitated cell viability (Figure 6(a)) and cell proliferation capacity (Figure 6(b)), but these effects have been weakened after PAWR expression was increased. The results from flow cytometry (Figure 6(c)), western blot (Figure 6(d)), and caspase-3 activity detection (Figure 6(e)) manifested that PAWR upregulation offsets the inhibitory regulation of miR-370-3p on cell apoptosis. Besides, the introduction of PAWR has reverted the miR-370-3p-mediated reduction of inflammatory cytokine release (Figures 6(f)–6(h)). The detection of MDA, ROS, and SOD demonstrated that miR-370-3p inhibited oxidative stress by targeting PAWR under the hypoxia condition (Figures 6(i)–6(k)). All in all, miR-370-3p exerted the protection for hypoxic AC16 cells via downregulating the expression of PAWR.

## 4. Discussion

circRNAs are characterized by covalently closed-loop structures that make circRNAs more stable to resist against RNA exonuclease than linear transcripts [16]. Our analysis indicated that circTRRAP was resistant to RNase R, and it was localized in the cytoplasm of cardiomyocytes. circRNAs have been implicated in the pathogenesis of various cardiovascular diseases, including AMI [17]. This research unraveled that circTRRAP promoted the hypoxia-induced cardiomyocyte dysfunction through the miR-370-3p-mediated PAWR upregulation.

CircRNA_000203 has aggravated the development of cardiac hypertrophy through elevating the level of Gata4 via inhibiting miR-26b-5p and miR-140-3p [18]. CircRNA_010567 exacerbated the myocardial fibrosis by sponging miR-141 leading to the downregulation of TGF-*β*1 [19]. CircMFACR modulated cell apoptosis in the heart through directly targeting miR-652-3p and indirectly upregulating MTP18 [20]. The functional experiments in this study revealed that silence of circTRRAP has resulted in the promotion of proliferation after cardiomyocytes were exposed to hypoxia. Apoptosis and inflammation are pivotal pathological aspects of AMI process [7]. The detection of apoptosis rate, apoptotic proteins, and caspase-3 activity demonstrated that circTRRAP knockdown suppressed cell apoptosis. The examination of inflammatory cytokines and oxidative indicators exhibited that the inflammatory response and oxidative stress induced by hypoxia were impeded when circTRRAP level was reduced. Significantly, the downregulation of circTRRAP could protect against the hypoxia-induced cell damages *in vitro*. It remains further investigation about the regulatory mechanism of circTRRAP.

Herein, we focused on the circRNA/miRNA/mRNA axis behind the function of circTRRAP in hypoxic cardiomyocytes. CircTRRAP was found to exert the sponge impact on miR-370-3p, and circTRRAP induced the negative change of miR-370-3p level. Moreover, miR-370-3p inhibitor has returned the si-circTRRAP-mediated protection for hypoxia-treated cardiomyocytes. Circ_0003204 was identified to inhibit endothelial cell injury in atherosclerosis by sponging miR-370-3p [21]. CircRNA_NEK6 retarded cell growth and invasion in thyroid cancer via the repression of miR-370-3p [22]. Our data suggested that circTRRAP could aggravate the hypoxia-induced dysfunction in cardiomyocytes by playing as a miR-370-3p sponge.

In addition, we validated that there was the target interaction between the 3′UTR of PAWR and miR-370-3p. Overexpression of miR-370-3p incurred the direct downregulation of PAWR expression, and circTRRAP exerted the positive effect on the level of PAWR through mediating the miR-370-3p expression. The correlation of PAWR and circRNA was few in human research. Song et al. asserted that circMTO1 reduced gastric cancer progression via sponging miR-199a-3p to increase the level of PAWR [23]. The regulatory function of circ_0068655 in AMI was associated with the miR-498-mediated expression promotion of PAWR [14]. What is more, miR-370-3p upregulation lightened the influences of hypoxia on cardiomyocytes by downregulating PAWR. The current evidence has provided the support for circTRRAP/miR-370-3p/PAWR axis participated in the hypoxia-induced damages of cardiomyocytes *in vitro*.

## 5. Conclusion

**Figure 7:**
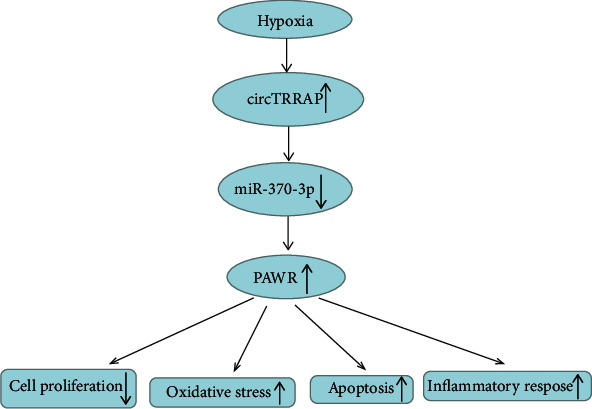
The graphical abstract of this study.

This study indicated that hypoxia treatment upregulated the expression of circTRRAP to promote the PAWR level via inhibiting miR-370-3p, then leading to the apoptotic, inflammatory, and oxidative damages in cardiomyocytes (Figure 7). CircTRRAP upregulation exacerbated cardiac injury, and circTRRAP inhibition could defend against cell dysfunction. CircTRRAP has the potential to act as a biomarker for AMI.

## Figures and Tables

**Table 1:** Primer sequences used for RT-qPCR.

Name	Primer sequences (5′-3′)
CircTRRAP	Forward: CTGCAAGCTCCTGCTGAAC Reverse: GGGGATCACTTGAGGGTTCT
TRRAP	Forward: GTGGACCTGTCTGAAGTCGTCA Reverse: TCACTTCCTGGGCAGAATCCAC
miR-370-3p	Forward: GCCGAGGCCTGCTGGGGTGG Reverse: CTCGTATCCAGTGCAGGGTC
GAPDH	Forward: GGTGAAGGTCGGAGTCAAC
	Reverse: AGAGTTAAAAGCAGCCCTGGTG
*β*-Actin	Forward: CACCATTGGCAATGAGCGGTTC Reverse: AGGTCTTTGCGGATGTCCACGT
U6	Forward: CTCGCTTCGGCAGCACA Reverse: AACGCTTCACGAATTTGCGT

## Data Availability

Data sharing not applicable to this article as no datasets were generated or analyzed during the current study.
